# *Curcuma longa* improves endothelial glycocalyx integrity and redox-inflammatory pathways in type 2 diabetes mellitus: a randomized double-blind placebo-controlled study

**DOI:** 10.1007/s00394-026-03963-3

**Published:** 2026-04-15

**Authors:** Drielly Rodrigues Viudes, Alanna Ramalho Mateus, Cristina Antoniali Silva, Maria do Carmo Franco

**Affiliations:** 1 Postgraduate Program in Translational Medicine, Department of Medicine, School of Medicine, Federal University of São Paulo (UNIFESP), São Paulo, SP Brazil; 2 School of Dentistry, Department of Basic Sciences, São Paulo State University (UNESP), Araçatuba, SP Brazil; 3 LiTiVasC - Laboratory of Translational Research in Vascular and Molecular Physiology, Physiology Department, School of Medicine, Federal University of São Paulo (UNIFESP), Rua Botucatu, 862 – 5° floor, São Paulo, SP 04023-062 Brazil

**Keywords:** *Curcuma longa*, Syndecans, Diabetic neuropathy, Nrf2, SIRT1, NF-κB p65

## Abstract

**Purpose:**

This randomized, double-blind, placebo-controlled trial investigated the effects of *Curcuma longa* extract (CLE) supplementation on vascular, redox-inflammatory biomarkers, and neuropathy symptoms in adults with type 2 diabetes mellitus (T2DM).

**Methods:**

Seventy-six adults with T2DM were randomized to receive CLE (1200 mg/day) or placebo for three months. Plasma concentrations of hyaluronic acid (HA), syndecan-1 (SDC1), syndecan-4 (SDC4), matrix metalloproteinases (MMP-2 and MMP-9), thioredoxin-1 (Trx1), thioredoxin-binding protein-2 (TBP2), sirtuin-1 (SIRT1), nuclear factor erythroid 2–related factor 2 (Nrf2), and the p65 subunit of nuclear factor kappa B (NF-κB p65) were measured at baseline and post-intervention. Neuropathy symptoms were assessed using a validated questionnaire.

**Results:**

Baseline characteristics were comparable between groups. After 3 months, CLE supplementation resulted in a significant reduction in neuropathy symptom score compared with placebo (*P* = 0.048). CLE significantly reduced markers of endothelial glycocalyx shedding, including SDC1 (66.5 vs. 113.0 ng/mL; *P* = 0.001), SDC4 (155.1 vs. 225.5 pg/mL; *P* = 0.040), and HA (259.8 vs. 371.7 ng/mL; *P* = 0.002), as well as MMP-2 (198.4 vs. 233.3 ng/mL; *P* < 0.001) and MMP-9 (1.2 vs. 2.0 ng/mL; *P* < 0.001). In parallel, CLE increased antioxidant/redox markers Trx1, SIRT1, and Nrf2 (all *P* < 0.05) and reduced TBP2 and NF-κB p65 levels (both *P* < 0.001), indicating coordinated modulation of vascular, redox, and inflammatory pathways.

**Conclusion:**

Short-term supplementation with CLE was associated with favorable modulation of vascular and redox-inflammatory biomarkers and improvement in neuropathy symptoms in adults with T2DM. These findings support the role of CLE as a promising bioactive nutritional supplement for the prevention and management of vascular and neuropathic complications associated with diabetes.

## Introduction

Type 2 Diabetes Mellitus (T2DM) is a chronic metabolic disorder characterized by persistent hyperglycemia, insulin resistance, and a state of chronic low-grade inflammation and oxidative stress [1, 2]. These alterations contribute substantially to the development and progression of microvascular and macrovascular complications, which represent major causes of morbidity and mortality among individuals with diabetes [3].

A critical determinant of vascular health is the endothelial glycocalyx (EG), a carbohydrate-rich layer that lines the luminal surface of endothelial cells [4]. The EG plays a central role in maintaining vascular integrity, regulating vascular permeability, mediating shear stress, and modulating inflammatory responses [4]. In T2DM, damage to the EG is common and results in increased shedding of its components into the circulation, thereby contributing to endothelial dysfunction and the development of vascular complications [5].

*Curcuma longa* has been extensively investigated for its diverse pharmacological properties, which are largely attributed to its principal bioactive compound, curcumin [6, 7]. These properties include potent antioxidant, anti-inflammatory, and antidiabetic effects [6, 7]. Although previous studies have demonstrated beneficial effects of curcumin on glycemic control, lipid profiles, and systemic inflammatory markers [8–10], its specific impact on biomarkers directly reflecting EG integrity and key intracellular redox and inflammatory signaling pathways in patients with T2DM has not yet been explored.

Therefore, this study aimed to evaluate the effects of *Curcuma longa* extract (CLE) supplementation on specific plasma biomarkers in adults with T2DM. The biomarkers assessed included components of the EG (syndecan-1, syndecan-4, and hyaluronic acid), matrix metalloproteinases involved in extracellular matrix remodeling (MMP-2 and MMP-9), and key redox-sensitive and inflammatory signaling molecules (thioredoxin-1, thioredoxin-binding protein-2, sirtuin-1, nuclear factor erythroid 2–related factor 2, and the p65 subunit of nuclear factor kappa B). By focusing on this integrated panel of biomarkers, the present study provides a better understanding of the mechanisms through which *Curcuma longa* may exert protective effects on endothelial health in the context of T2DM.

## Materials and methods

### Study design

This was a randomized double-blind placebo-controlled clinical trial, that used a convenience sampling method to recruit participants. The study followed the consolidated statement of reporting trials (CONSORT) guidelines and was approved by the local ethics committee (approval number: 5.554.571). Informed consent was obtained from all individual participants included in the study. The study was registered with the International Clinical Trials Registration Platform (ICTRP) via Brazilian Clinical Trials Registry (ReBEC) (approval number: RBR-3qqr4yk).

### Participants

Participants were enlisted from the basic health unit outpatient located in Birigui (São Paulo, Brazil) from January 2023 to June 2024. Participants of both sexes aged between 40 and 70 years old that met the diagnostic criteria of T2DM were included in the study [11]. The participants were excluded: (i) pregnant women; (ii) lactating women; (iii) individuals with difficulty walking and/or who are unable to step onto the platform scale; (iv) current or recent use of antibiotics and anti-inflammatories (3 months); (v) who are using or have recently used (in the last 6 months) *Curcuma longa* in the form of encapsulated powder, dry plant extract, plant tincture or other pharmaceutical presentation; (vi) the same criteria were applied to the use of antioxidant supplements or multivitamins and minerals; (vii) participants using antiplatelet agents, anticoagulants, low molecular weight heparin and thrombolytic agents; (viii) participants with autoimmune, inflammatory and infectious diseases, chronic kidney disease, gastric ulcers, liver disease, cancer and acquired immunodeficiency syndrome (AIDS). These exclusion criteria were designed to reduce potential confounding from conditions or treatments known to independently influence endothelial function, oxidative stress, inflammation, neuropathy outcomes, or could affect the effects of *Curcuma longa* supplementation.

### Randomization and blinding

We randomized the participants to receive either *Curcuma longa* extract (CLE) supplementation or placebo. A randomization list was generated by an external person not related to the study electronically on random.org, in blocks of two with a 1:1 ratio. The randomization system allowed only one randomization per patient. Intervention was blinded for participants, healthcare teams, investigators, data collectors, and statisticians. To ensure blinding, CLE and placebo were provided in identical, opaque capsules with the same dark orange color, packaged in identical plastic containers, and labeled as capsule “X” and capsule “Y.” Capsules were tightly sealed and had no detectable odor, preventing identification of the intervention based on visual appearance or smell. Both were produced and labeled by Estratti Vegetali compounding pharmacy (São Paulo, Brazil).

### Intervention and study protocol

Participants in the CLE group received 1200 mg/day by consuming four 300 mg capsules of standardized dry extract of *Curcuma longa* with 97% curcuminoids orally after each main meal in two doses for 3 months. This dose was based on a previously published study, which have demonstrated biological efficacy and safety of curcuminoid supplementation within a dose range of approximately 500–2000 mg/day [12, 13]. Participants allocated to the placebo group received identical capsules containing an inert substance, administered similarly. Moreover, all participants were instructed to maintain their habitual diet during the intervention.

Adherence to supplementation was primarily assessed through capsule counts performed at each study visit, combined with structured interviews regarding daily intake and missed doses. Participants were instructed to return all unused capsules, allowing objective estimation of compliance. In addition, standardized records of supplement dispensing were maintained. WhatsApp messaging was used solely as a supportive communication tool to reinforce adherence and clarify participant questions, and not as the primary method for compliance assessment. Supplementation was discontinued in the event of adverse effects, including gastrointestinal symptoms (e.g., stomach pain, vomiting, or diarrhea), or upon withdrawal of informed consent for personal reasons.

### Primary and clinical outcomes

The primary outcome was the plasmatic change of syndecan-1 (SDC1), syndecan-4, (SDC4) hyaluronic acid (HA), matrix metalloproteinases (MMP-2, MMP-9), thioredoxin-1 (Trx1), thioredoxin-binding protein-2 (TBP2), sirtuin-1 (SIRT1), nuclear factor erythroid 2–related factor 2 (Nrf2), and the p65 subunit of nuclear factor kappa B (NF-κB p65). Clinical outcome included changes in score of diabetic neuropathy. Glycemic control parameters were not included as primary outcomes.

### Anthropometric, neuropathy symptom, and physical activity assessment

Height was measured by a stadiometer to the nearest 0.5 cm while the participants wore no shoes. The body weight was measured using calibrated digital platform scale while wearing light clothes and no shoes. The waist circumference was measured at the midpoint between the lower rib margin and the iliac crest at the end of normal expiration. BMI was calculated as weight (kg) divided by height (m^2^). All anthropometrics parameters were measured at baseline and end of the study by the same researcher. The assessment of neuropathic symptoms was carried out using the neuropathy symptom score while short form of International Physical Activity Questionnaire (IPAQ) was used to measure participants physical activity at the baseline and end of study. The reliability and validity of these questionnaire were validated for Brazilian Portuguese [14, 15].

### Blood sampling and biochemical assay

Venous blood samples were taken at the baseline and after 3 months of intervention. EDTA blood samples were separated by centrifugation at 2500 rpm for 15 min at 4 °C to obtain plasma and were stored at − 80 °C until analyses. All plasmatic biomarkers levels were quantified using commercially available ELISA kits validated for human plasma samples (SDC1, SDC4, Trx1, SIRT1, Nrf2 from Abcam, Cambridge, United Kingdom; HA, TBP2, NF-κB p65 from Elabscience, Houston, USA). Assays were performed according to the manufacturers’ protocols, with reported detection limits and acceptable intra- and inter-assay coefficients of variation. Baseline and post-intervention samples from the same participant were analyzed within the same assay run to reduce inter-assay variability. Samples were analyzed in duplicate, and laboratory personnel were blinded to treatment allocation.

### Statistical analysis

Statistical analyses were conducted using SPSS version 21.0 for Windows (IBM Corporation, USA). All analyses were conducted on an intention-to-treat basis, assessing statistically significant differences between the means of the two groups at baseline and 3 months, separately. Categorical data were described by percentage and analyzed using chi-square test. Continuous variables were assessed for normality using the Shapiro–Wilk test and expressed as median with interquartile range [IQR]. A nonparametric Mann–Whitney test was used to compare plasmatic levels of biomarker between placebo and CLE group at baseline and after 3 months of intervention. In order to evaluate the outcome parameters at baseline and after 3 months of intervention in the same group were performed Wilcoxon test for paired samples. The spearman’s correlation coefficient was performed to evaluate the relationship of the delta change between independent variables. In addition, the effect was reported using the Hodges–Lehmann estimator with 95% confidence intervals (CI). Statistical tests were two-tailed and the significance level was set at *P* < 0.05.

## Results

### Participant characteristics and adherence

**Fig. 1:**
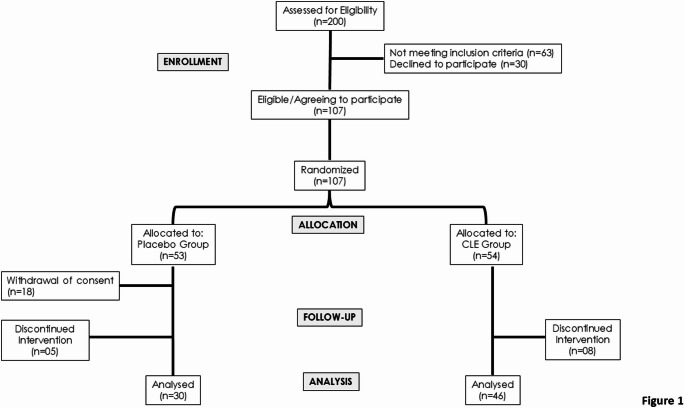
CONSORT flowchart. Enrollment, allocation, follow-up, and analysis. Lost to follow-up due to withdrew consent (n = 18) and discontinued intervention (n = 13). Final analysis was made in 76 participants. CLE: *Curcuma longa* extract

Between January 2023 to June 2024, 200 participants with T2DM were assessed for eligibility. Of these, 93 were excluded, due to not meeting inclusion criteria (n = 63) or declined to participate (n = 30). Consequently, 107 participants were randomized, with 54 in the CLE group and 53 in the placebo group. Eighteen participants in the placebo group withdrew consent, resulting in 89 participants included. During follow-up, five participants in the placebo group and eight in the CLE group discontinued intervention due to adverse event, such as stomach pain, vomiting, or diarrhea, leading to 76 participants being included in the analysis. Follow-up for the last participant was completed in June 2024. The study flowchart is provided in Fig. 1.

The adherence rate (number of total capsule intake divided by the total number of capsules made available for 3 months) was 90.5% in the CLE group and 88.4% in the placebo group.

### Baseline data

At baseline time, there were no significant differences between the groups regarding age, sex distribution, diabetes duration or neuropathy symptom score (Tables 1, 2). Anthropometric parameters, including BMI, waist circumference, and hip circumference, were also comparable between the groups (Table 1). Additionally, no statistically significant differences were found in, nutritional status, comorbidities, medication use, diabetes-related complications, smoking status, or physical activity levels (Table 1). Moreover, baseline levels of plasmatic endothelial glycocalyx (EG) biomarkers, as well as inflammatory and oxidative stress-related markers, were similar between groups (Table 2).

**Table 1:** Baseline demographic, anthropometric, and clinical characteristics of participants with type 2 diabetes mellitus in the placebo and *Curcuma longa* extract (CLE) groups

Variables	Placebo (n = 30)	CLE (n = 46)	*P* value
Age (years)	58.0 [8.0]	59.0 [13.0]	0.386
*Sex* (%)			
Male Female	36.7 63.3	28.3 71.7	0.441
Diabetes duration (years)	4.0 [9.0]	6.5 [12.0]	0.277
IMC (kg/m^2^)	32.8 [7.0]	31.3 [9.0]	0.476
Waist circumference (cm)	109.8 [15.6]	108.0 [17.3]	0.176
Hip circumference (cm)	102.0 [14.4]	96.0 [16.7]	0.673
*Nutritional status* (%)			
Healthy weight Overweight Obesity	6.7 50 43.3	15.2 52.2 32.6	0.426
*Comorbidities *(%)			
Arterial hypertension Osteoarthritis	66.7 26.7	54.3 10.9	0.285 0.074
*Medication use* (%)			
Oral hyperglycemic agents Insulin SGLT2 inhibitors DPP-4 inhibitors β blocker Calcium blocker Diuretics ACE inhibitor AT_1_ antagonist Antilipemics agents	100 30 26.7 6.7 16.7 6.7 23.3 3.3 60 26.7	97.8 30.4 19.6 6.5 23.9 10.9 23.9 6.5 41.3 41.3	0.416 0.968 0.466 0.980 0.449 0.536 0.954 0.543 0.111 0.192
*Diabetes complications* (%)			
Retinopathies Others	30 6.7	26.1 6.5	0.929
*Smoking status* (%)			
Yes No	3.3 96.7	2.2 97.8	0.758
*IPAQ* (%)			
Sedentary behavior … Irregularly active Active	26.7 46.7 26.7	17.4 52.2 30.4	0.625

Values are expressed as median [IQR] for continuous variables and percentage (%) for categorical variables. Between-group comparisons were performed using Mann–Whitney U test for continuous variables and the chi-square test for categorical variables

BMI, body mass index; NSS: Neuropathy Symptom Score; IPAQ, International Physical Activity Questionnaire; ACE, angiotensin-converting enzyme; DPP-4, dipeptidyl peptidase-4; SGLT2, sodium-glucose co-transporter 2

**Table 2:** Changes in NSS score, endothelial glycocalyx, inflammatory, and oxidative stress-related plasma biomarkers in participants from the placebo and *Curcuma longa* extract (CLE) groups at baseline (0 months) and after 3 months of intervention

Outcome	Groups	Baseline	3 months	Within-group p	Between-group p	Δ HL (95%CI)	^#^HL (95%CI)
*Neuropathy*							
NSS score (points)	Placebo CLE	2.5 [6.0] 3.0 [6.0]	2.5 [5.0] 0 [3.5]	0.250 **0.001**	0.872 **0.048**	0 (0 to 0) − 1.5 (− 3.0 to 0.7)	0 (0 to 2)
*Endothelial glycocalyx integrity*							
SDC1 (ng/mL)	Placebo CLE	84.5 [59.9] 85.2 [56.1]	113.0 [93.3] 66.5 [38.4]	0.144 ** < 0.001**	0.675 **0.001**	8.5 (− 2.5 to 33.3) − 15.2 (− 24.7 to –9.4)	36.1 (11.9 to 64.6)
SDC4 (pg/mL)	Placebo CLE	250.8 [203.2] 206.7 [302.0]	225.5 [151.5] 155.1 [264.3]	0.090 ** < 0.001**	0.832 **0.040**	4.5 (− 26.9 to 5.2) − 46.0 (− 72.3 to –30.8)	65.0 (13.6 to 102.6)
HA (ng/mL)	Placebo CLE	369.1 [186.0] 347.4 [271.4]	371.7 [180.9] 259.8 [138.9]	0.106 **0.004**	0.694 **0.002**	2.4 (− 0.4 to 5.8) − 68.1 (− 106.4 to − 22.0)	98.1 (38.4 to 118.7)
*Extracellular matrix remodeling*							
MMP-2 (ng/mL)	Placebo CLE	224.5 [52.7] 230.1 [69.8]	233.3 [60.9] 198.4 [60.3]	0.178 ** < 0.001**	0.262 ** < 0.001**	2.9 (− 1.5 to 9.4) − 32.1 (− 49.5 to − 19.5)	41.2 (21.5 to 66.2)
MMP-9 (ng/mL)	Placebo CLE	1.5 [1.4] 2.0 [1.5]	2.0 [1.3] 1.2 [1.0]	0.092 ** < 0.001**	0.285 ** < 0.001**	0.3 (− 0.1 to 0.6) − 0.9 (− 1.2 to − 0.6)	0.9 (0.5 to 1.3)
TIMP-1 (ng/mL)	Placebo CLE	46.6 [7.5] 44.1 [9.3]	47.6 [6.6] 45.3 [12.6]	0.168 0.062	0.148 0.473	1.5 (− 0.8 to 3.1) 2.6 (− 0.1 to 5.5)	1.3 (− 2.6 to 5.1)
TIMP-2 (ng/mL)	Placebo CLE	122.2 [24.5] 120.3 [26.8]	122.0 [20.1] 123.1 [36.5]	0.877 0.723	0.942 0.603	0.4 (− 4.9 to 4.6) 2.0 (-8.2 to 11.0)	− 2.5 (− 11.9 to 6.0)
*Redox/inflammatory signaling*							
Trx1 (ng/mL)	Placebo CLE	40.6 [9.8] 35.7 [12.9]	41.2 [10.9] 44.4 [16.0]	0.581 ** < 0.001**	0.129 **0.022**	0.4 (− 0.8 to 1.5) 9.1 (6.6 to 12.7)	− 6.2 (− 11.4 to − 1.0)
TBP2 (ng/mL)	Placebo CLE	2.8 [1.0] 3.2 [1.1]	2.9 [1.3] 2.1 [1.0]	0.548 ** < 0.001**	0.129 ** < 0.001**	0.1 (− 0.1 to 0.2) − 1.2 (− 1.4 to − 0.9)	0.8 (0.5 to 1.2)
SIRT1 (ng/mL)	Placebo CLE	1.5 [0.7] 1.6 [0.7]	1.6 [0.9] 2.1 [0.6]	0.118 **0.001**	0.506 **0.001**	0.1 (− 0.1 to 0.3) 0.5 (0.3 to 0.6)	− 0.5 (− 0.8 to − 0.3)
Nrf2 (pg/mL)	Placebo CLE	127.2 [61.8] 130.0 [109.4]	141.2 [48.9] 170.8 [110.1]	0.334 **0.002**	0.610 ** < 0.001**	− 2.2 (− 8.8 to 1.4) 36.7 (14.7 to 55.2)	− 33.9 (− 67.5 to − 5.5)
NF-κB p65 (ng/mL)	Placebo CLE	2.0 [0.6] 2.3 [0.5]	2.1 [0.6] 1.8 [0.4]	0.225 ** < 0.001**	0.175 ** < 0.001**	0.1 (− 0.1 to 0.3) − 0.4 (− 0.5 to − 0.3)	0.4 (0.2 to 0.5)

Data are presented as median [IQR]. Between-intervention groups comparisons were assessed using the Mann–Whitney U test. Paired comparisons were assessed using Wilcoxon test. ^#^HL (95%CI): Hodges–Lehmann estimate (CLE–Placebo) representing the median difference in outcomes between groups with corresponding 95% confidence intervals; ΔHL (95%CI): Hodges–Lehmann median difference (3 months − baseline; negative indicates a decrease) with corresponding 95% confidence intervals

SDC1, syndecan-1; SDC4, syndecan-4; HA, hyaluronic acid; MMP-2, matrix metalloproteinase-2; MMP-9, matrix metalloproteinase-9; TIMP-1/2, tissue inhibitor of metalloproteinases-1/2; Trx1, thioredoxin-1; TBP2, thioredoxin-binding protein-2; SIRT1, sirtuin-1; Nrf2, nuclear factor erythroid 2–related factor 2; NF-κB p65, nuclear factor kappa B p65 subunit

p value in bold indicate significant comparison between the groups

### CLE intervention data

**Fig. 2:**
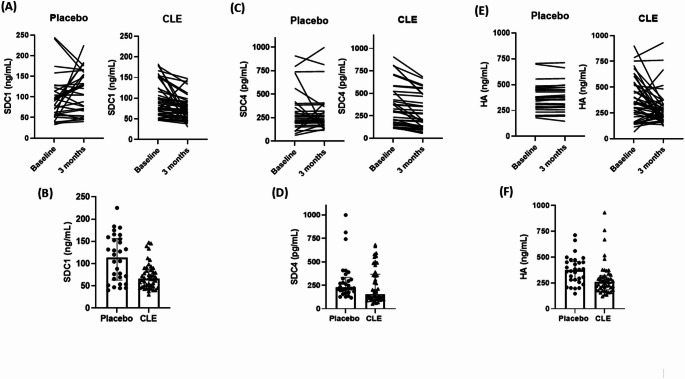
Effects of Curcuma longa supplementation on endothelial glycocalyx integrity biomarkers in patients with type 2 diabetes. Individual data at baseline and after the 3-months placebo or CLE intervention on circulating levels of **A** SDC1, **C** SDC4, and **E** HA. Bar charts illustrate median, interquartile range, and individual values of plasma concentrations of **B** SDC1, **D** SDC4, and **F** HA after 3 months in both placebo and CLE groups

**Fig. 3:**
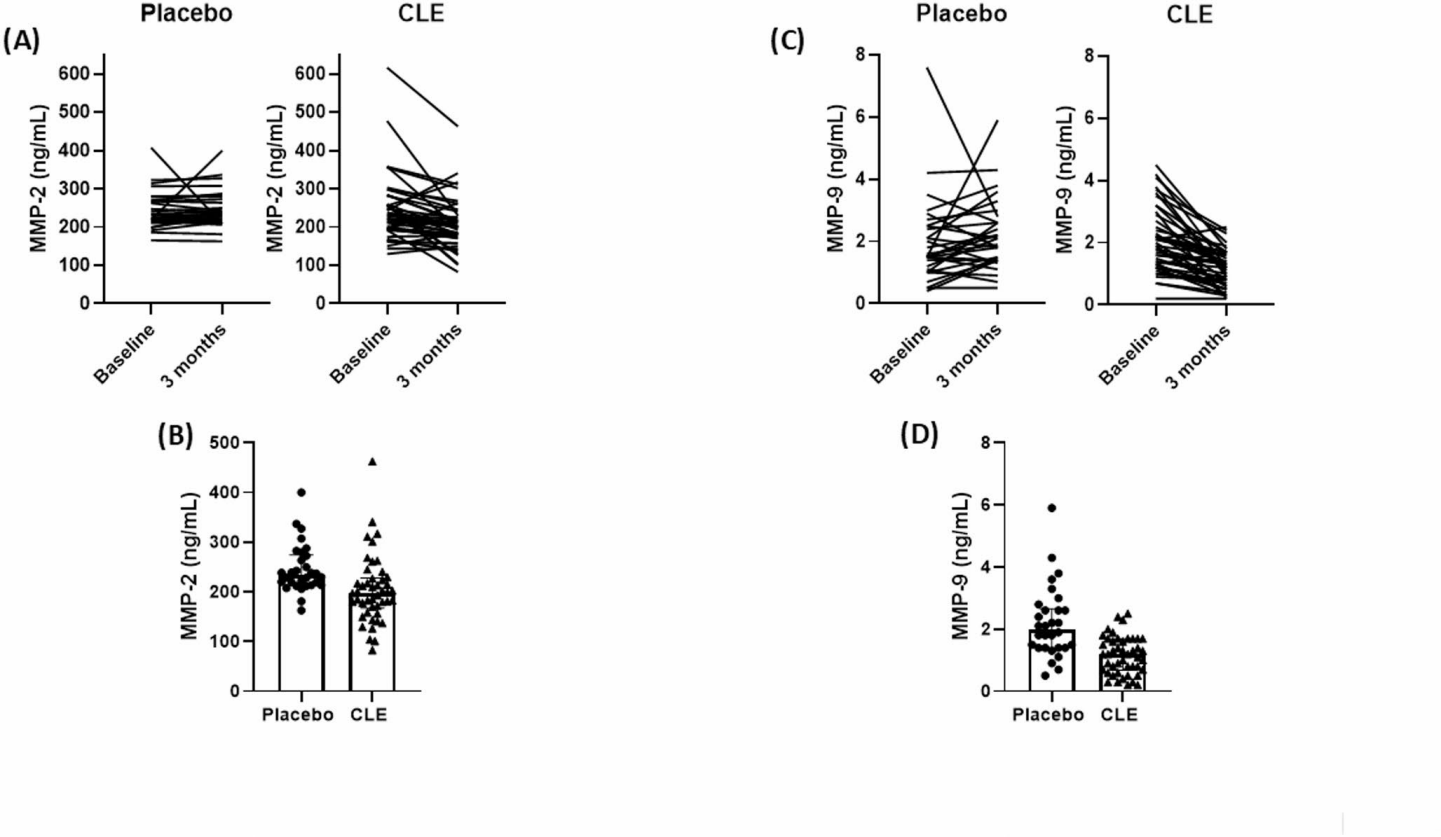
Effects of Curcuma longa supplementation on extracellular matrix remodeling biomarkers in patients with type 2 diabetes. Individual data at baseline and after the 3-months placebo or CLE intervention on circulating levels of **A** MMP-2 and **C** MMP-9. Bar charts illustrate median, interquartile range, and individual values of plasma concentrations of **B** MMP-2 and **D** MMP-9 after 3 months in both placebo and CLE groups

After 3 months of intervention, participants in the CLE group exhibited a significant reduction in plasma levels of SDC1, SDC4, and HA compared to those in the placebo group (Table 2; Fig. 2). This improvement in EG biomarkers was accompanied by lower circulating levels of both MMP-2 and MMP-9 in the CLE group (all *p* < 0.001) relative to the placebo group (Table 2; Fig. 3). However, no significant differences were observed between groups regarding plasma concentrations of TIMP-1 and TIMP-2 (Table 2).

**Fig. 4:**
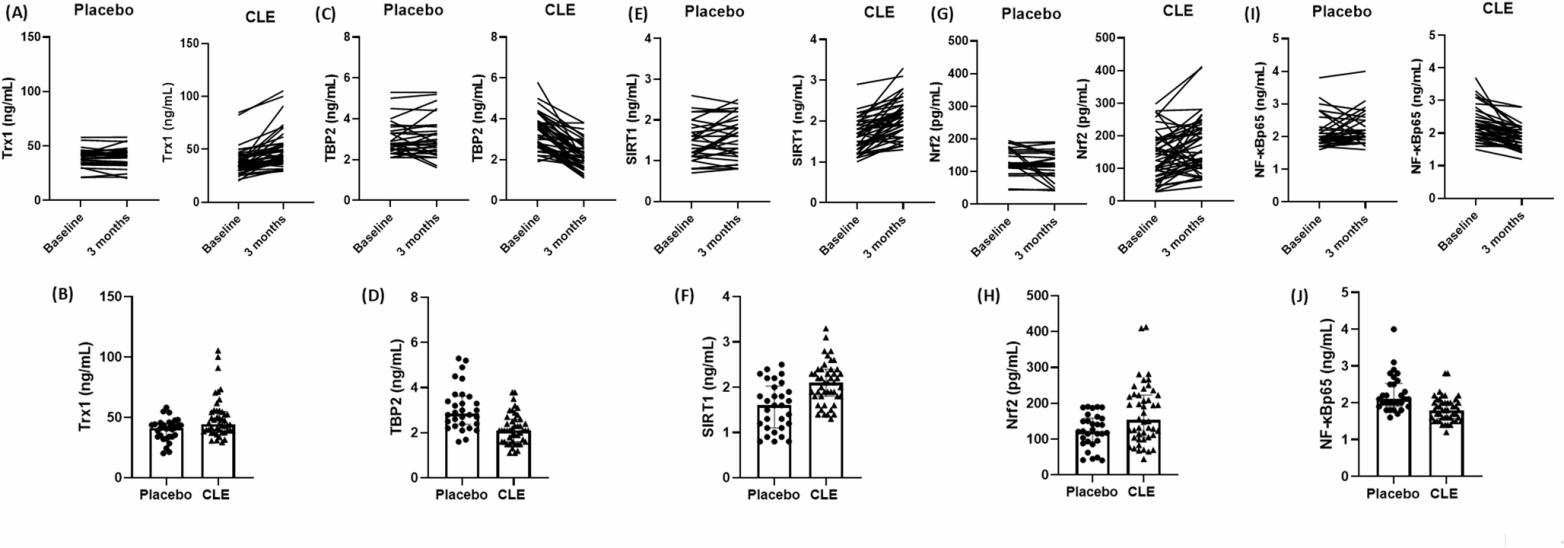
Effects of Curcuma longa supplementation on Redox/inflammatory signaling in patients with type 2 diabetes. Individual data at baseline and after the 3-months placebo or CLE intervention on circulating levels of **A** Trx1, **C** TBP2, **E** SIRT1, **G** Nrf2, and **I** NF-κB p65 subunit. Bar charts illustrate median, interquartile range, and individual values of plasma concentrations of **B** Trx1, **D** TBP2, **F** SIRT1, **H** Nrf2, and **J** NF-κB p65 subunit

Notably, plasma levels of Trx1 were significantly higher in the CLE group compared to the placebo group after 3 months of supplementation. This was accompanied by a reduction in TBP2 concentrations in CLE-treated individuals with T2DM (Table 2; Fig. 4). Another important finding was that CLE supplementation also increased circulating levels of both SIRT1 and Nrf2 (Table 2; Fig. 4). Conversely, the levels of the NF-κB p65 were significantly decreased in the CLE group compared to the placebo group (Table 2; Fig. 4). Considering clinical outcome, CLE-treated participants reported significant improvement of the neuropathy symptom score than those treated with placebo (Table 2).

### Correlation between delta change of the plasma biomarkers of EG integrity, matrix remodeling, and redox-inflammatory

Weak-to-moderate correlations were observed between changes of the several biomarkers. Delta change in plasmatic levels of HA correlated with delta of the TBP2 (*rho* = 0.265, *P* = 0.021) and NF-κB p65 (*rho* = 0.253, *P* = 0.028), whereas delta change in SDC1 was correlated with delta change in MMP-9 (*rho* = 0.347, *P* = 0.002), Trx1 (*rho* = -0.338, *P* = 0.003), TBP2 (*rho* = 0.251, *P* = 0.029), SIRT1 (*rho* = − 0.300, *P* = 0.008), and NF-κB p65 (*rho* = 0.303, *P* = 0.008). Moreover, delta changes in plasmatic levels of SDC4 also correlated with delta changes in MMP-9 (*rho* = 0.290, *P* = 0.011), Trx1 (*rho* = − 0.400, *P* < 0.001), TBP2 (*rho* = 0.365, *P* = 0.001), SIRT1 (*rho* = -0.294, *P* = 0.010), and NF-κB p65 (*rho* = 0.256, *P* = 0.025).

Delta changes in plasmatic levels of MMP-2 correlated with delta change in TBP2 (*rho* = 0.411, *P* < 0.001), and Nrf2 (*rho* = − 0.294, *P* = 0.010), while delta change in MMP-9 levels were correlated with delta change in Trx1 (*rho* = − 0.417, *P* < 0.001), TBP2 (*rho* = 0.338, *P* = 0.003), SIRT1 (*rho* = − 0.308, *P* = 0.007), Nrf2 (*rho* = − 0.398, *P* < 0.001), and NF-κB p65 (*rho* = 0.413, *P* < 0.001).

Spearman’s correlation analysis revealed significant associations between delta change of the Trx1 with the delta change of TBP2 (*rho* = − 0.568, *P* < 0.001), SIRT1 (*rho* = 0.330, *P* = 0.004), Nrf2 (*rho* = 0.417, *P* < 0.001), and NF-κB p65 (*rho* = − 0.455, *P* < 0.001). The delta change of TBP2 was also correlated with the delta change of these biomarkers (SIRT1: *rho* = − 0.345, *P* = 0.002; Nrf2: *rho* = − 0.407, *P* < 0.001; NF-κB p65: *rho* = 0.348, *P* = 0.002). Additionally, delta change in SIRT1 was positively correlated with delta change in Nrf2 (*rho* = 0.273, *P* = 0.017) and inverse with delta change in the NF-κB p65 (*rho* = − 0.425, *P* < 0.001). Also, significant inverse correlation was observed between delta changes of the Nrf2 and NF-κB p65 (*rho* = -0.308, *P* = 0.007).

### Correlation between delta changes of the neuropathy symptom score with plasma biomarkers

Weak correlations were observed between delta change in neuropathy symptom score with delta of the plasmatic levels of SDC4 (*rho* = 0.319, *P* = 0.005), MMP-9 (*rho* = 0.286, *P* = 0.001), TBP2 (*rho* = 0.291, *P* = 0.001), and Nrf2 (*rho* = -0.226, *P* = 0.049), indicating modest but consistent associations between biomarker modulation and clinical outcomes.

## Discussion

To our knowledge, this is the first study in patients with T2DM to demonstrate coordinated modulation of EG components, matrix remodeling enzymes, and redox-inflammatory pathways following *Curcuma longa* supplementation.

Damage to the EG represents an early and critical event in the pathogenesis of vascular complications in T2DM [5]. The EG is a dynamic layer composed of proteoglycans, glycoproteins, and glycosaminoglycans that serves as a functional interface between circulating blood and the vascular endothelium [4]. Under diabetic conditions, persistent hyperglycemia and oxidative stress accelerate enzymatic degradation and shedding of EG constituents, leading to increased vascular permeability and endothelial dysfunction [5].

In the present study, the significant reductions in circulating levels of SDC1, SDC4, and HA after three months of CLE supplementation suggest a protective effect on glycocalyx integrity. Experimental evidence supports this interpretation. In vitro studies have demonstrated that curcumin attenuates glycocalyx shedding by downregulating heparanase expression, an enzyme directly involved in EG degradation [16]. Curcumin has also been shown to reduce damage to heparan sulfate and SDC1, further supporting direct preservation of glycocalyx structure [17].

Beyond EG preservation, CLE markedly reduced plasma levels of MMP-2 and MMP-9, enzymes critically involved in extracellular matrix turnover. Increased MMP activity contributes to vascular remodeling, enhanced permeability, and progression of diabetic complications [18]. These findings are consistent with experimental models showing that curcuminoids suppress metalloproteinase expression in diabetes-related conditions [19]. Notably, the positive correlations between delta changes in MMP-9 and both SDC1 and SDC4 support the concept that reduced MMP activity contributes to endothelial integrity preservation. The absence of significant changes in TIMP-1 and TIMP-2 suggests that CLE effects on MMP activity are likely mediated through modulation of inflammatory and oxidative signaling rather than direct regulation of tissue inhibitors.

A key finding of this study was the significant increase in plasma levels of Trx1, SIRT1, and Nrf2 in the CLE group, accompanied by reductions in TBP2 and NF-κB p65. The concomitant increase in Trx1 and decrease in TBP2 indicate enhanced thioredoxin system activity. Trx1 is a central antioxidant protein involved in scavenging reactive oxygen species (ROS), repairing oxidatively damaged proteins, and regulating redox-sensitive signaling pathways [20]. In contrast, TBP2 acts as a negative regulator of Trx1, and its downregulation is associated with improved antioxidant capacity and cellular protection against oxidative injury [20]. The inverse correlation between delta changes in Trx1 and TBP2 observed in our study reinforces the notion that CLE promotes a favorable redox environment through coordinated regulation of this system.

The increase in Nrf2 levels further supports activation of endogenous cytoprotective responses. Nrf2 regulates the transcription of numerous antioxidant enzymes, counteracting oxidative stress, a key driver of T2DM pathogenesis and complications [21]. Curcumin-induced Nrf2 activation has been consistently associated with reduced oxidative stress, improved insulin signaling, and attenuation of diabetic complications [21, 22]. Importantly, Nrf2 activation suppresses TBP2 expression, creating a feedback mechanism that enhances Trx1 availability and activity [22]. Together, these findings provide a mechanistic explanation for the observed redox improvements following CLE supplementation. By enhancing Trx1, reducing TBP2, and upregulating Nrf2, *Curcuma longa* supplementation appears to restore redox balance and attenuate one of the central pathogenic drivers of vascular dysfunction and tissue damage in T2DM.

The significant elevation of SIRT1 highlights its central role in cellular defense against metabolic stress [23]. The correlations between changes in SIRT1, TBP2, and Nrf2 suggest coordinated regulation through a SIRT1–Nrf2–TBP2 axis. SIRT1 promotes Nrf2 stabilization and nuclear translocation via deacetylation of Nrf2 and/or its inhibitor Keap1, while Nrf2 suppresses TBP2 transcription and induces antioxidant gene expression, resulting in an integrated antioxidant response [24].

SIRT1 also exerts potent anti-inflammatory effects through deacetylation and inhibition of the NF-κB p65 subunit, reducing transcription of pro-inflammatory cytokines and adhesion molecules [25]. This mechanism is supported by the inverse correlation observed between SIRT1 and NF-κB p65 levels in our study. Additionally, SIRT1 suppresses MMP activity by inhibiting NF-κB-dependent transcription and stabilizing the extracellular matrix through modulation of TGF-β/Smad signaling [26]. The inverse association between changes in SIRT1 and MMP-9 provides further mechanistic insight into how CLE may protect the endothelial glycocalyx and attenuate vascular remodeling in T2DM.

Consistent with these observations, plasma levels of NF-κB p65 were significantly reduced in the CLE group, confirming attenuation of inflammatory signaling. NF-κB is an important regulator of innate immune responses, and chronic activation of its p65 subunit is strongly implicated in T2DM-related vascular inflammation, endothelial dysfunction, and matrix degradation [27, 28]. Therefore, the present findings emphasize the integrated suppression of NF-κB activity within a broader redox-inflammatory network. Curcumin is known to inhibit NF-κB activation through multiple mechanisms. It prevents the phosphorylation and degradation of IκBα, thereby retaining NF-κB in the cytoplasm and blocking its nuclear translocation [29]. Furthermore, curcumin directly impairs the DNA-binding activity of the p65 subunit by modulating redox-sensitive cysteine residues and suppresses upstream kinases such as IKKβ and Akt [30].

The observed negative correlations between delta changes in NF-κB p65 and Trx1, SIRT1, and Nrf2 further illustrate coordinated regulation of oxidative stress and inflammation. Trx1 suppresses NF-κB activation by reducing critical thiol groups in p65 and preventing its DNA binding [31]. SIRT1 exerts a potent inhibitory effect by reducing its transcriptional activity and inflammatory output [32]. Nrf2 activation also indirectly inhibits NF-κB by enhancing antioxidant defenses and reducing the levels of ROS [33]. Conversely, positive correlations with TBP2 and HA reflect associations with cellular stress and endothelial injury. Together, these relationships support the hypothesis that CLE exerts vascular-protective effects by rebalancing redox-inflammatory signaling rather than targeting isolated pathways.

From a clinical perspective, the significant improvement in neuropathy symptom scores in the CLE group adds functional relevance to these molecular findings. The correlations between changes in neuropathy symptoms and alterations in SDC4, MMP-9, TBP2, and Nrf2 suggest that improvements in endothelial integrity, redox balance, and inflammatory status may contribute to symptomatic relief. While these associations do not establish causality, they provide clinically meaningful support for the biological relevance of CLE supplementation in T2DM.

### Strengths and limitations

This study has some strengths. First, it was designed as a randomized, double-blind, placebo-controlled clinical trial, which minimizes bias and strengthens the internal validity of the findings. Second, the intervention was based on a standardized *Curcuma longa* extract with high curcuminoid content, ensuring reproducibility and consistency of the nutritional supplement. Third, we assessed a comprehensive panel of mechanistic biomarkers, including endothelial glycocalyx components, matrix metalloproteinases, and redox-inflammatory regulators such as Trx1, TBP2, SIRT1, Nrf2, and NF-κB p65. This broad approach allowed us to provide integrated insights into the molecular pathways modulated by *Curcuma longa* in type 2 diabetes. In addition, the inclusion of a clinical outcome (e.g. neuropathy symptom score) provided translational relevance by linking biochemical improvements to patient-reported benefits.

Several limitations of this study should be acknowledged. First, although the sample size was sufficient to detect significant changes in circulating biomarkers and neuropathy symptom scores, the study was not designed to evaluate long-term clinical outcomes (e.g. neuropathy progression, microvascular complications, quality of life), as well as do not establish direct mechanistic causality. In addition, dietary intake was not formally controlled or recorded, which may represent a source of residual confounding. Also, glycemic control markers, including HbA1c and fasting plasma glucose, were not assessed, which limits conclusions regarding the metabolic effects of *Curcuma longa* supplementation on glucose homeostasis.

Neuropathy assessment relied on a validated symptom questionnaire; however, objective neurophysiological measurements, such as nerve conduction studies or quantitative sensory testing, were not performed. This may limit the ability to comprehensively characterize neuropathy severity and disease progression. Furthermore, plasma curcumin concentrations and pharmacokinetic parameters were not measured, precluding evaluation of bioavailability and interindividual variability in response to supplementation.

The use of strict exclusion criteria, while strengthening internal validity by minimizing biological and pharmacological confounding, may have reduced the generalizability of the findings to patients with more complex comorbidity profiles. Additionally, the relatively short intervention period of three months limits conclusions regarding the long-term sustainability of the observed molecular and clinical effects.

Finally, participant recruitment was based on convenience sampling from a single clinical center, which may further restrict extrapolation of the results to broader or more diverse populations. Future studies with longer follow-up periods, objective neuropathy assessments, inclusion of glycemic control outcomes, and multicenter recruitment strategies are warranted to confirm and extend these findings.

## Conclusion

In conclusion, this randomized, double-blind, placebo-controlled trial provides the first clinical evidence in patients with T2DM that short-term supplementation with a standardized *Curcuma longa* extract is associated with modulation of interconnected vascular, redox, and inflammatory pathways. CLE supplementation was linked to improvements in markers of endothelial glycocalyx integrity, attenuation of matrix remodeling, and activation of antioxidant and anti-inflammatory signaling involving the Trx1/TBP2 system, SIRT1, Nrf2, and NF-κB.

These molecular changes were accompanied by clinically relevant improvements in patient-reported neuropathy symptoms, supporting the biological plausibility and potential nutritional relevance of *Curcuma longa* as a bioactive food component. However, given the short intervention period and the absence of long-term clinical endpoints, the findings should be interpreted as indicative of early biological and symptomatic responses rather than definitive evidence of sustained clinical benefit.

Overall, the results suggest that *Curcuma longa* supplementation may represent a promising adjunctive nutritional strategy for modulating pathways implicated in vascular dysfunction and neuropathic symptoms in diabetes. Future studies with longer follow-up, broader and more diverse populations, and objective clinical outcomes are needed to determine the durability and clinical significance of these effects and to further define the role of *Curcuma longa* in the nutritional management of chronic diseases.

## Data Availability

The data that supports the findings of this study are available from the corresponding author upon reasonable request.
